# Cytomegalovirus cell tropism and clinicopathological characteristics in gastrointestinal tract of patients with HIV/AIDS

**DOI:** 10.1186/s13000-022-01193-9

**Published:** 2022-01-14

**Authors:** Lei Sun, Jia-min Chen, Kun Yang, Liang Zhang, Zhi-yuan Ma, Xiang-mei Chen, Man Li, Xingang Zhou, Ping Li, Hong-xin Zhao, Jiang Xiao, Li-ming Qi, Peng Wang

**Affiliations:** 1 Department of Pathology, Beijing Ditan Hospital, Capital Medical University, No. 8 Jing Shun East Street, Chaoyang District, Beijing, 100015 People’s Republic of China; 2 Department of Gastroenterology, Beijing Ditan Hospital, Capital Medical University, Beijing, 100015 China; 3 Center for Infectious Diseases, Beijing Ditan Hospital, Captial Medical University, Beijing, 100015 China

**Keywords:** HIV/AIDS, CMV, Cell tropism, Digestive tract, PDGFRα, Nrp2, Clinicopathological features

## Abstract

**Background:**

Cytomegalovirus (CMV) has been recognized as one of the frequently occurring opportunistic infections (OIs) reported in the patients having human immunodeficiency virus/acquired immunodeficiency syndrome (HIV/AIDS). In addition, it has been identified as the factor leading to gastrointestinal (GI) tract disorder among HIV/AIDS population. CMV exhibits broad cell tropism in different organs. This study evaluated the CMV cell tropism and clinicopathological characteristics of CMV infection in the different GI regions in HIV/AIDS cases.

**Methods:**

Using nucleic acid in situ hybridization (ISH), CMV was detected in the gastrointestinal mucosal biopsy samples. The paraffin-embedded samples were stained with hematoxylin and eosin (HE) and immunohistochemistry (IHC), respectively.

**Results:**

A total of 32 HIV/AIDS patients were enrolled in this study. Fourteen of these patients underwent gastroscopy, while the remaining eighteen received colonoscopy. CMV-infected cells were observed at 46 GI sites. Among them, the colon was the region with the highest susceptibility to GI CMV infection (*n* = 12, 26.1%). The CMV giant cell inclusion bodies were detected in epithelial cells and mesenchymal cells, including histiocytes, smooth muscle cells, fibroblasts, and endothelial cells. In the duodenum, there were markedly more positive epithelial cells than mesenchymal cells (*p* = 0.033). In contrast, in the esophagus (*p* = 0.030), cardia (*p* = 0.003), rectum (*p* = 0.019), colon (*p* < 0.001), and cecum (*p* < 0.001), there were notably less positive epithelial cells than mesenchymal cells. The expression levels of PDGFRα and Nrp2 in the mesenchymal cells were higher than the epithelial cells in cardia, cecum, colon, sigmoid, and rectum, especially in the areas with ulcers. However, Nrp2 in the epithelial cells was higher than that in the duodenum. Moreover, the positive CMV DNA in peripheral blood was related to the CMV-positive cell count, as well as the ulceration in GI tract (*p* = 0.035 and 0.036, respectively).

**Conclusions:**

The colon has been identified as the GI site with the highest susceptibility to CMV infection. There are different CMV-infected cells in the different sites of the GI that relate to the expression level of PDGFRα and Nrp2. CMV DNA positive in the blood is related to the positive CMV cell count, as well as ulceration in the GI tract.

## Background

CMV is among the most common pathogen-induced opportunistic infections detected in patients with infections resulting from the human immunodeficiency virus/acquired immunodeficiency syndrome (HIV/AIDS) [1]. Among such cases, gastrointestinal (GI) tract is among those most susceptible organs to CMV infection. GI manifestations take up about 10% CMV disorders among AIDS cases [2]. CMV infection may be detected in any region of the GI tract, from the mouth to the rectum [3]. Typically, endoscopy, together with mucosal biopsy, is most frequently used for diagnosis, and CMV colitis is confirmed via pathology through identifying the virus-induced cytopathic effects on biopsy tissue. CMV exhibits a broad cell tropism in different organs. The cells, most frequently identified as permissive to CMV, include epithelial cells, smooth muscle cells, endothelial cells, and fibroblasts. Over the years, many different cell surface proteins and molecules have been reported to function as CMV entry receptors, especially platelet-derived growth factor receptor alpha (PDGFRα) and Neuropilin2 (Nrp2), they mediated CMV into fibroblasts and endothelial/epithelial cells, respectively [4]. CMV tropism may be related to the expression of these cell surface receptors. The cell tropism and clinicopathological features for GI CMV infection have been rarely investigated in the literature. Therefore, this work was carried out aiming to assess CMV cell tropism in GI tract, and the pathological features as well as a clinicopathological correlation for CMV disorder were also evaluated.

## Materials and methods

### Patients

This study enrolled altogether 32 HIV/AIDS patients with GI CMV infection from Beijing Ditan Hospital from January 2010 to January 2020. All patients enrolled in this study underwent gastroscopy or colonoscopy to evaluate the GI symptoms, including abdominal pain, diarrhea, and hemafecia. Clinical data of all cases were acquired based on patient charts using the electronic medical record system in our center. Those factors extracted included age of patient, gender, serum CD4+ T cells, CD8+ T cells, leukocyte, erythrocyte, hemoglobin, platelet, HIV viral loads, CMV viral loads, and follow-up biopsies. Written informed consent was obtained from the participants by physicians. The study protocol was approved by the Ethics Committee of Beijing Ditan Hospital, Capital Medical University. All procedures performed in studies involving human participants were in accordance with the ethical standards and with the 1964 Helsinki declaration and its later amendments or comparable ethical standards.

### Acquisition of mucosal tissue specimens from GI tract

As part of the procedure, mucosal tissues were sampled from inflammatory foci in the esophagus, cardia, gastric antrum, gastric body, duodenum, cecum, colon, sigmoid and rectum, as well as from regions that appeared to be unaffected. The extracted tissues were then fixed in 10% formaldehyde solution. Subsequently, serial sections (4 μm) of tissues fixed in formalin and embedded within paraffin were stained with HE, acid-fast, hexamine silver, periodic acid-Schiff and IHC. Nucleic acid ISH was adopted to prepare samples for detection of CMV.

### EnVision two-step method for immunohistochemical staining

Embedded sections were deparaffinized with xylene followed by an alcohol gradient and water rinses and incubated with 0.3% hydrogen peroxide for 10 min at room temperature to eliminate endogenous peroxidase activity. After antigen retrieval under high pressure with a citrate buffer, individual slides were incubated at 4 °C overnight with mouse anti-human PDGFRα (Beijing Zhongshan Biotechnology, Lot:ZA0377, 1:40 dilution), mouse anti-human Nrp2 (Abcam Company, Lot:GR3249327–7, 1:200 dilution), Slides were then washed three times with phosphate-buffered saline (PBS), followed by horseradish peroxidase-labeled secondary antibodies (37 °C for 30 min). Slides were then washed and developed with DAB, hematoxylin counterstained, and mounted. PBS diluent was used in place of individual primary antibodies as negative controls.

### Nucleic acid ISH of CMV

Nucleic acid ISH using the digoxigenin-labeled probes was conducted in accordance with the manufacturer protocols. CMV probe was diluted at 1:50–1:100, which generated a strong brown-to-yellow positive signal. The hybridization kit equipped with the digoxigenin probe for detecting CMV, as well as the nitroblue tetrazolium (NBT)/5-bromo-4-chloro-3-indolyl phosphate (BCIP) assay kit, was provided by Leica Biosystems (CMV Probe:REF:PB0614, Lot:62163).

### Semi-quantitative evaluation of CMV DNA in mucosal tissues from GI tract

Ten regions of the lamina propria were selected at random for microscopic evaluation. Under high magnification (Nikon 80i, × 400), a grid counter was used to count the CMV-positive cells. The mean positive cell count in each high power field (HPF) was calculated, and then these cases were divided into three groups, including positive cells< 5 /HPF, 5–10/HPF, > 10 /HPF.

### Statistical analysis

SPSS 20.0 (IBM statistics, SPSS, Chicago, IL) was adopted for all statistical tests. Fisher’s exact test and Student’s t-test were employed to evaluate the differences. The chi-square test was utilized to analyze those categorical variables. For continuous values, they were expressed in the manner of mean ± standard deviation (SD). A difference of *p* < 0.05 indicated statistical significance.

## Results

### Features of patients

29 out of the 32 patients with HIV/AIDS enrolled in this study were males, while 3 were females, with age ranging 25 to 58 years (average, 38.0 ± 10.0 years). In these patients, 14 underwent gastroscopy to check the upper GI tract, whereas the rest 18 received colonoscopy to examine the lower GI tract. CMV infection was confirmed by biopsy and ISH in all patients, but CMV DNA in peripheral blood was detected only in 18 patients, including 7 undergoing gastroscopy and 11 receiving colonoscopy. The difference was not statistically significant between these two groups (*p* = 0.530). In addition, CD4 cell count of all patients dropped to below 200 cells/μL, and 27 patients had the CD4 cell count of < 50 cells/μL. Patient demographic characteristics and other laboratory measurements, including serum CD4+ T cells, CD8+ T cells, leukocyte, erythrocyte, hemoglobin, platelet, and HIV viral loads, displayed no significant difference between patients receiving gastroscopy and those undergoing colonoscopy. Data are presented in Table 1.

**Table 1:** Demographics and laboratory parameters of patients with gastroscopy and colonoscopy

	Upper Gastrointestinal Tract(*n* = 14)	Lower Gastrointestinal Tract (*n* = 18)	Total(*n* = 32)	*P* value
Sex				
Male	13 (92.9%)	16 (88.9%)	29	0.702
Female	1 (7.1%)	2 (11.1%)	3	
Average Age	38.6 ± 8.4	39.3 ± 11.3	38.0 ± 10.0	0.862
Serum CD4+ T cells	19.2 ± 22.9	37.9 ± 43.2	29.3 ± 35.9	0.174
Serum CD8+ T cells	449.6 ± 335.7	576.6 ± 553.1	517.6 ± 461.4	0.478
Leukocyte	4.56 ± 2.18	5.74 ± 4.84	5.19 ± 3.82	0.390
Erythrocyte	3.23 ± 0.46	3.11 ± 0.79	3.16 ± 0.65	0.629
Hemoglobin	98.2 ± 14.8	92.8 ± 26.8	95.3 ± 21.9	0.516
Platelet	185.2 ± 58.3	188.3 ± 130.8	186.9 ± 101.8	0.934
HIV viral load	201,674 ± 190,958	253,520 ± 257,044	228,724 ± 224,303	0.592
Patients number of serum CMV DNA positive	7 (38.9%)	11 (61.1%)	18	0.530

### Pathological findings and CMV cell tropism in mucosal biopsies from HIV/AIDS patients with CMV infection

In these 32 patients, the CMV-infected cells were observed at 46 sites of the GI tract. Colon was the GI site exhibiting the highest susceptibility to CMV disorder (*n* = 12, 26.1%). Meanwhile, the stomach (*n* = 10, 21.7%), including the cardia (*n* = 3, 6.5%), antrum (*n* = 5, 10.9%) and gastric body (*n* = 2, 4.3%), was the second most frequently affected site, followed by esophagus (*n* = 7, 15.2%), cecum (*n* = 7, 15.2%), duodenum (*n* = 4, 8.7%), sigmoid (*n* = 3, 6.5%) and rectum (*n* = 3, 6.5%) (Table 2).

**Table 2:** cellular localization of CMV in gastrointestinal tract mucosal biopsies

Sites(*n* = 46)	Mesenchymal cells/HPF	epithelial cells/HPF	*P* value
Esophagus(*n* = 7, 15.2%)	9.8 ± 11.8	3.2 ± 5.6	0.030
Cardia(*n* = 3, 6.5%)	7.3 ± 4.6	1.0 ± 1.8	0.003
gastric antrum(*n* = 5, 10.9%)	8.3 ± 8.8	8.2 ± 11.5	0.976
gastric body(*n* = 2, 4.3%)	7.2 ± 4.0	9.0 ± 3.7	0.478
Duodenum(*n* = 4, 8.7%)	1.0 ± 2.2	17.4 ± 20.6	0.033
Cecum(*n* = 7, 15.2%)	18.5 ± 18.9	0.1 ± 0.4	0.000
Colon(*n* = 12, 26.1%)	10.5 ± 10.6	0.2 ± 0.7	0.000
sigmoid(*n* = 3, 6.5%)	8.0 ± 10.3	0	–
rectum(*n* = 3, 6.5%)	6.7 ± 6.1	1.1 ± 2.0	0.019

**Fig. 1:**
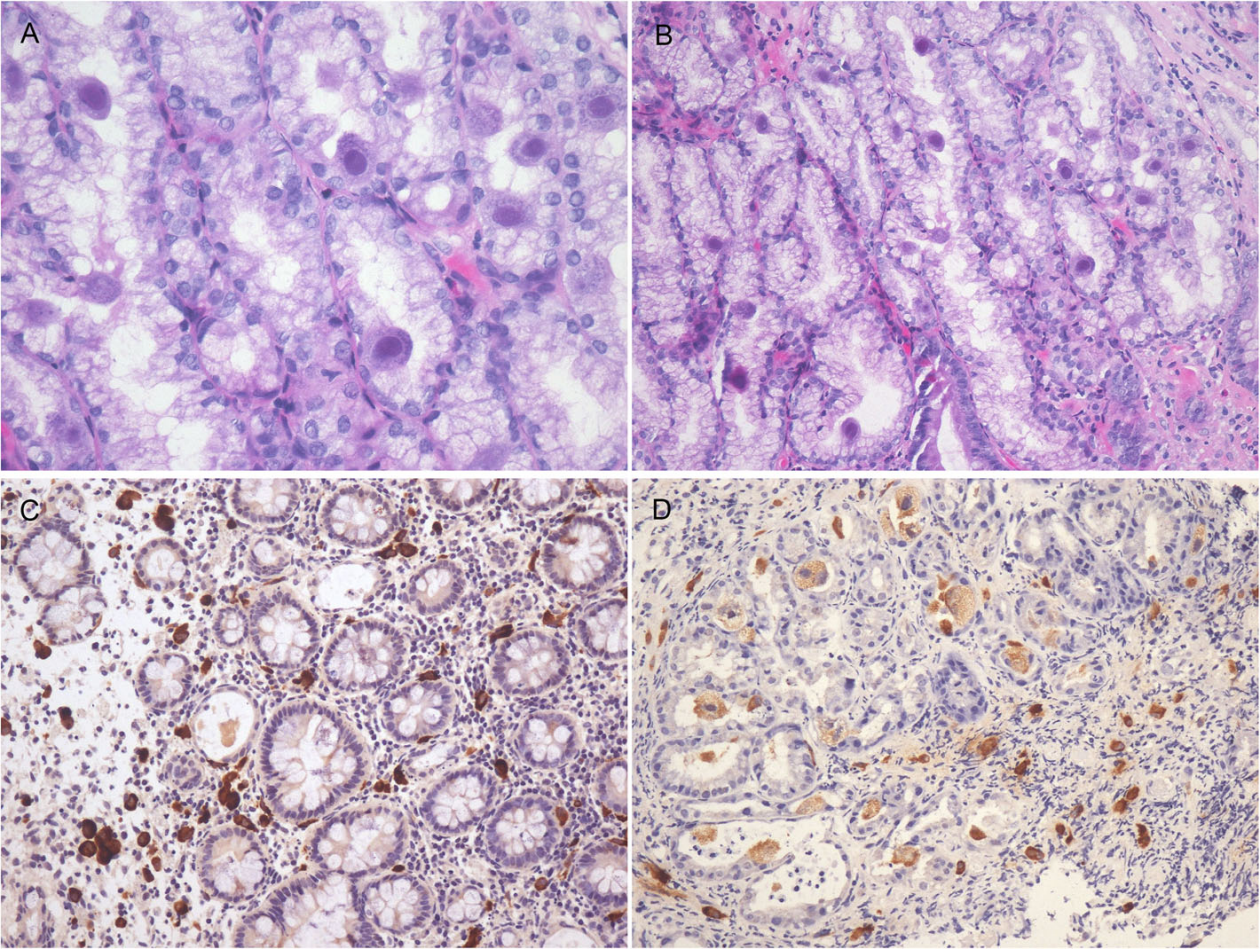
CMV cell tropism in gastrointestinal tract from patients with HIV/AIDS. (**A**) CMV viral inclusions showed an ‘eagle eye’ appearance in some virus-infected cells, other CMV inclusions were characterized by abundant coarse eosinophilic cytoplasmic inclusions within enlarged cells. HE, original magnification 400×. (**B**) CMV mostly infected epithelial cells in duodenum. HE, original magnification 200×. (**C**) CMV mostly infected mesenchymal cells in colon (brown). ISH, original magnification 200×. (**D**) Epithelial cells and mesenchymal cells can be all infected by CMV in gastric antrum. ISH, original magnification 200 ×

Characteristics of the biopsied intestinal mucosal samples included edema, congestion, lymphocytosis, and rare lymphoid aggregates. 27 of the 32 enrolled cases (84.4%) developed obvious active (neutrophilic) as well as chronic (lymphoplasmacytic) inflammation; besides, some areas of ulceration were detected in 14 cases. Only 5 cases showed mild inflammation. HE staining and nucleic acid ISH for CMV revealed that giant cell inclusion bodies were observed in epithelial cells and mesenchymal cells, including histiocytes, smooth muscle cells, fibroblasts, and endothelial cells. Those inclusion bodies were round or oval, and were surrounded by a distinct air halo, a characteristic also known as the “owl’s eye.” Other CMV inclusions were characterized by the affluent rough eosinophilic inclusions of cytoplasm inside those enlarged cells in the absence of clear inclusions of nucleus (Fig.1a).

In the duodenum, CMV most frequently infected the epithelial cells, and there were significantly more positive epithelial cells than mesenchymal cells(*p* = 0.033) (Fig.1b). In the esophagus (*p* = 0.030), cardia (*p* = 0.003), rectum (*p* = 0.019), colon (*p*<0.001), and cecum (*p*<0.001), there were markedly less positive epithelial cells than mesenchymal cells (Fig.1c). In the sigmoid, no epithelial cell was positive. In the gastric antrum and gastric body, both epithelial cells and mesenchymal cells were infected by CMV (Fig.1d), and the difference between these two types of cells was not statistically significant (Table 2).

### Expression of PDGFRα and Nrp2 in the different sites of the GI tract

**Fig. 2:**
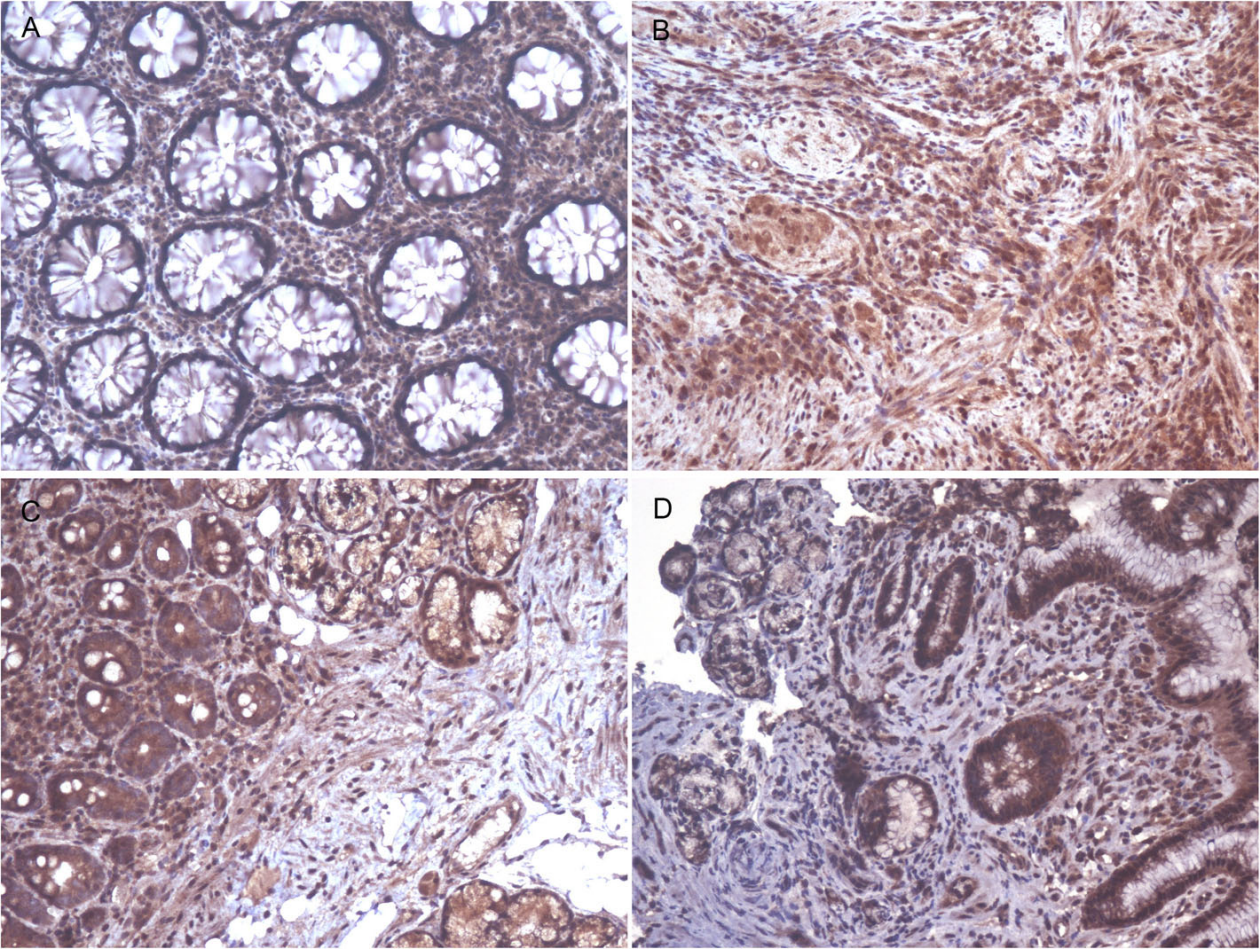
Expression of PDGFRα and Nrp2 in different sites of the gastrointestinal tract in patients with HIV/AIDS and CMV. (**A**) Expression level of PDGFRα on mesenchymal cells was higher than epithelial cells in colon. IHC, original magnification 200×. (**B**) Expression of Nrp2 on hyperplastic mesenchymal cells in areas with ulcers in colon. IHC, original magnification 200×. (**C**) Expression level of Nrp2 on epithelial cells was higher than mesenchymal cells in duodenum. IHC, original magnification 200×. (**D**) Expression level of PDGFRα on epithelial cells and mesenchymal cells was similarly in the gastric antrum. IHC, original magnification 200 ×

Immunoreactive PDGFRα and Nrp2 were detected in the cell membranes and within the cytoplasm of epithelial cells and mesenchymal cells, including histiocytes, fibroblasts, smooth muscle cells, and endothelial cells. However, the immunohistochemical expression intensity and cell type were distinct in the different sites of the GI tract. The expression levels of PDGFRα and Nrp2 in the mesenchymal cells were higher than that in the epithelial cells in cardia, cecum, colon, sigmoid, and rectum, especially in areas with ulcers (Fig.2a and Fig.2b). But Nrp2 in epithelial cells was higher in the duodenum (Fig.2c). In the gastric antrum and gastric body, both epithelial cells and mesenchymal cells expressed PDGFRα and Nrp2 similarly (Fig.2d). These expression characteristics were roughly consistent with the CMV cell tropism in the GI tract.

### Clinicopathological correlations of CMV infection in GI tract

According to the mean CMV-positive cell count in every HPF, we divided the 32 enrolled cases into 3 groups: positive cells<5 /HPF, 5–10/HPF, >10 /HPF. Among the 8 cases with CMV positive cells< 5 /HPF, the blood CMV DNA was detectable only within 2 patients, whereas in the 12 cases with positive cells> 10 /HPF, blood CMV DNA was detectable within 10 patients. In the 14 patients with ulceration in GI tract, 11 were CMV DNA positive in blood; by contrast, in the 18 cases with no ulceration, only 7 were CMV DNA positive in blood. Upon Fisher’s exact test, CMV DNA positive in blood was related to the CMV-positive cell count, as well as the ulceration in GI tract (*p* = 0.035 and 0.036, respectively) (Table 3).

**Table 3:** Clinicopathological correlation of gastrointestinal tract CMV infection

		Number of CMV positive cells/HPF			*P* value	Pathological changes		*P* value	Total
		<5	5–10	>10		Ulcer(+)	Ulcer(−)		
CMV DNA in blood	(+)	2	6	10	0.035	11	7	0.036	18
	(−)	6	6	2		3	11		14
	Total	8	12	12		14	18		32

## Discussion

CMV is a double-stranded DNA virus, which is one of the β-herpesvirus family members. The virus is widespread among the general population, and the CMV seroprevalence may be > 60% [5]. CMV infection can be initiated through close contact, intravenous injection, blood transfusion, sexual intercourse, placental transmission, or organ transplantation. CMV disorder is an opportunistic infection that may threaten human life, and it is frequently seen among HIV/AIDS patients. Typically, GI CMV infection is one of the most common clinical manifestations, which represents a severe complication that affects HIV/AIDS patients; besides, it is the primary factor leading to mortality and morbidity [6]. In this article, we discussed the CMV cell tropism in GI tract, pathological features and clinicopathological correlations of GI CMV disorder.

Among cases infected with HIV, the susceptibility to symptomatic disorder is related to the immunosuppression degree, which elevates markedly at the case of number of CD4 cells < 200 cells/μL. According to one study, CMV infection is remarkably prevalent in HIV-infected patients who have the number of CD4 cells < 50 cells/μL, compared with those having the number of CD4 cells> 50 cells/μL [7]. Similar results were obtained in this study, the CD4 cell number in all patients dropped to < 200 cells/μL, among them, 27 cases had that of < 50 cells/μL.

CMV can affect any GI site, among which, colon is the most susceptible site. Among GI CMV patients, colon involvement has been commonly detected, which accounts for 94% patients [8, 9]. Colon was also the most commonly affected site in our results, which accounted for 26.1%. Stomach was most susceptible to CMV infection in upper GI tract, including cardia, antrum and gastric body in 10 patients. Similar results were also noted by Bonetti et al. [10]. But another study finds that, esophagus ranks the second place in terms of its susceptibility to CMV infection in the GI tract, which is only second to colon [11]. CMV-induced esophagitis was found in 7 cases in our study, only second to the stomach. CMV duodenitis is exceedingly rare, which is found in only 1 out of 30 cases with duodenum involvement, as reported in one study [10]. There were 4 cases with CMV duodenitis identified in our study. Patient demographic characteristics and other laboratory measurements, including serum CD4+ T cells, CD8+ T cells, leukocyte, erythrocyte, hemoglobin, platelet, and HIV viral loads, were not significantly different in CMV infection between the upper and lower GI tracts.

Several methods, including viral culture, serological tests, polymerase chain reaction (PCR), and shell viral assay, are proposed to diagnose GI CMV infection. However, virologic and serologic tests may be restricted in diagnosing the GI-restricted CMV disorder [12]. The primary process for diagnosis is endoscopy for identifying mucosal lesion, as well as tissue biopsy to confirm the infection. CMV infection is histopathologically diagnosed according to those virus-infected cells in the biopsied tissues (namely, the viral cytopathic effect). Typically, these virus-infected cells show the characteristic intracellular inclusions, with an ‘owl’s eye’ appearance on slides subjected to routine HE staining, or IHC detection of CMV viral inclusions [13, 14]. Histological findings have been shown with a sensitivity of 93% and a specificity of 100% in confirming the diagnosis [15]. However, it is impossible that all viral inclusions are identified by HE staining alone, because the complex inflammatory background may mask the inclusions, or the “atypical” viral inclusion may be characterized by the affluent rough eosinophilic inclusions of cytoplasm in those enlarged cells with no distinct inclusions of nucleus, as seen in our cases. Furthermore, IHC may yield ambiguous results for various reasons, it is also not the approach with the highest sensitivity in CMV detection. It remains controversial about the necessity of the routine implementation of CMV IHC in biopsy with moderate-severe inflammation [16]. PCR is a sensitive, specific, and rapid molecular tool that may be helpful to aid in early diagnosis of CMV infection on equivocal or clinically highly suspicious small GI biopsies [17], but it cannot show the position of positive cells in situ. Therefore, ISH was applied in this study to detect CMV in GI tract.

Notably, the significant active inflammation, especially with ulceration, stands for a vital clue to suspect GI CMV infection. 27 of our enrolled cases showed significant inflammation, and 14 had ulceration. Nonetheless, CMV infection can not be completely excluded when there is no distinct inflammation. For instance, the inflammation was not significant in 5 of our cases. The less inflammation background may be related to the Highly Active Antiretroviral Therapy (HAART) after CMV infection, or the absence of inflammation may represent a sampling error, specimens were taken from near the lesion [6]. Ulceration was detected in 14 cases in our study, with the deepening of ulcer, vascular erosion might result in substantial bloody diarrhea [18]. The progression of CMV disorder is associated with ulcer formation in the GI tract [13]. We demonstrated that, CMV DNA positive in blood was correlated with ulceration in the GI tract. In addition, if ulcers were observed in the GI tract, the CMV DNA in blood was more likely to be positive.

CMV demonstrates broad tropism in the GI tract. The CMV-infected cell types involve epithelial cells and mesenchymal cells, including histiocytes, endothelial cells fibroblasts and smooth muscle cells. However, we found that, the main types of CMV-infected cells were different at the diverse locations of GI tract. In the duodenum, CMV most frequently infects epithelial cells, whereas mesenchymal cells are the primary target in the esophagus, cardia, cecum, colon and rectum. In the stomach, both epithelial cells and mesenchymal cells can be infected by CMV. The cell tropism may relate to the viral entry machinery. Genetic analysis showed that CMV cell tropism require products of viral genes UL128L, together with gH/gL, forming the gH/gL/pUL128L pentamer complex (PC) that is required for the infection of epithelial or endothelial cells, whereas gH/gL and gO form the gH/gL/gOtrimer complex (TC), required for the infection of both cell types [19]. In 2016, a receptor for the TC that mediates entry into fibroblasts was identified as PDGFRα [20], while in 2018, a receptor for the PC that mediates entry into endothelial/epithelial cells was identified as neuropilin2 (Nrp2) [21]. Therefore, we detected the two receptors in different cell types in GI tract. We found that PDGFRα and Nrp2 all expressed in the epithelial cells and mesenchymal cells, and the expression levels of PDGFRα and Nrp2 in the mesenchymal cells were higher than those in the epithelial cells in cardia, cecum, colon, sigmoid, and rectum, especially in areas with ulcers. These may be the reason for markedly more positive mesenchymal cells than epithelial cells in these sites. The results of some studies were similar to ours. A flow cytometry analysis showed that an Nrp2 receptor was expressed on the cell surface of both epithelial/endothelial cells and fibroblasts. Thus, fibroblasts expressed similar levels of PDGFRα and Nrp2, which made the fibroblasts more susceptible to CMV infection compared to the epithelial cells [4].

Those affected cells become larger, which can comprise the cytoplasmic and nuclear inclusions. The nuclear inclusions are amphophilic, which are encompassed via a rarified chromatin zone, leading to the appearance of “owl’s eye”, while the cytoplasmic inclusions are eosinophilic and granule-like. Because the owl’s eye sign is not often seen in daily clinical practice, ISH serves as the most efficient approach when HE staining is unable to identify the inclusions, particularly when CMV infection is clinically suspected or the endoscopic results of HIV/AIDS cases are abnormal [6].

Peripheral blood CMV DNA test at molecular level is extensively utilized for monitoring those cases susceptible to infection [22]. According to some studies, the viral load of CMV shows poor association with CMV infection burden within GI tissues [23, 24]. Meanwhile, it is indicated in another study that, cell number found in the biopsied GI tissues is related to the systemic CMV viremia [16]. In our study, we also demonstrated that, positive CMV DNA in blood was related to the CMV-positive cell number within the GI tract. Therefore, we speculated that, the lower CMV-infected cell number led to the higher probabilities of negative blood CMV DNA and as a “bystander”; in addition, a higher number of CMV-infected cells resulted in the higher chances of positive blood CMV DNA and a systemic disorder that induces pathological changes, like ulceration or serious inflammation observed in the biopsied tissues. Nonetheless, it’s not uncommon to have isolated GI CMV disease without detectable CMV DNA, as observed among 14 of our cases.

## Conclusions

In conclusion, this study is carried out mainly to explore the CMV cell tropism and correlations among clinicopathological features of GI CMV infection. The results suggest that different CMV-infected cells in the different sites of the GI relating to the expression level of PDGFRα and Nrp2 as well as the vital part of pathological assessment in diagnosing and managing CMV among HIV/AIDS cases. Endoscopy is recommended to obtain a tissue biopsy for HE staining and ISH among HIV/AIDS patients with GI tract symptoms, so as to confirm the CMV infection.

## Data Availability

The datasets used and/or analyzed during the current study available from the corresponding author on reasonable request.

## Abbreviations

CMV: Cytomegalovirus
HIV: Human Immunodeficiency Virus
AIDS: Acquired Immunodeficiency Syndrome
GI: gastrointestinal
IHC: immunohistochemical
HE: haematoxylin and eosin
ISH: in situ hybridization
PDGFRα: platelet-derived growth factor receptor alpha
Nrp2: Neuropilin2
